# Differential enrichment of H3K9me3 in intrahepatic cholangiocarcinoma

**DOI:** 10.1186/s12920-022-01338-1

**Published:** 2022-08-26

**Authors:** Sheng Hu, Xuejun Wang, Tao Wang, Lianmin Wang, Lixin Liu, Wenjun Ren, Xiaoyong Liu, Weihan Zhang, Weiran Liao, Zhoujun Liao, Renchao Zou, Xiaowen Zhang

**Affiliations:** 1 Department of Hepatobiliary Surgery, The Second Affiliated Hospital of Kunming Medical University, No. 374, Dianmain Road, Kunming, China; 2 Department of Cardiovascular Surgery, The First People’s Hospital of Yunnan Province, Kunming, China; 3 Department of Thoracic Surgery, The Second Affiliated Hospital of Kunming Medical University, Kunming, China; 4 Department of Cardiology, The Second Affiliated Hospital of Kunming Medical University, Kunming, China

**Keywords:** Cholangiocarcinoma, H3K9me3, WNT signaling pathway, Gene expression

## Abstract

**Background:**

Intrahepatic cholangiocarcinoma (ICC) is a malignant tumor, which poses a serious threat to human health. Histone 3 lysine 9 trimethylation (H3K9me3) is a post-translational modification involved in regulating a broad range of biological processes and has been considered as potential therapeutic target in types of cancer. However, there is limited research on investigating profiles of histone modification H3K9me3 in ICC patients.

**Methods:**

In this study, we applied the ChIP-seq technique to investigate the effect of H3K9me3 on ICC. Anti-H3K9me3 antibody was used for ChIP-seq in ICC (RBE cell lines) and HIBEpic (normal cell lines). MACS2 (peak-calling tools) was then used to identify the peaks recorded in RBE and HIBEpic cell lines. Gene expression, mutation and clinical data were downloaded from TCGA and cBioPortal databases.

**Results:**

H3K9me3 exhibited abnormal methylation and influenced the process of abnormal gene expression in patients suffering from ICC. The Wnt/β-Catenin signaling pathway (also known as simply the WNT signaling pathway) was enriched in H3K9me3-regulated genes.

**Conclusions:**

We are the first to report that H3K9me3 may play an important role in the progression of ICC. It promotes the understanding of epigenetic molecular mechanisms for ICC.

**Supplementary Information:**

The online version contains supplementary material available at 10.1186/s12920-022-01338-1.

## Background

Cholangiocarcinoma (CCA) is a life-threatening malignancy. Based on the anatomical location, CCA can be classified as intrahepatic cholangiocarcinoma (ICC), perihilar cholangiocarcinoma (PCCA), or distal cholangiocarcinoma (DCCA). The incidence and case-fatality rates are increasing worldwide [1–3]. ICC is one of the most common malignant tumor arising from the liver, and it makes up about 10% of all CCA. The poor outcome can be attributed to the insidious and aggressive nature of ICC[4, 5]. Effective treatment methods are urgently needed to be developed [6]. It is believed that the development and progression of ICC are multifactorial and multistep pathological processes involving oncogene activation, inactivation of tumor suppressor genes, tumor metastasis, apoptosis and cell cycle dysregulation, and tumor genetics and epigenetic alterations [3]. It has been reported that multi-omics data analysis was performed and the results revealed that most of the genetic variations observed in patients suffering from ICC could be attributed to copy number variations (CNV) [7, 8]. The aberrantly expressed genes were highly expressed [9]. The specific mechanism among them is still unclear.

Histones are structural proteins present in chromosomes that associate with DNA and form nucleosomes. In mammals, based on the molecular weights, histones are classified as H1, H3, H2A, H2B, and H4. H3 is the most extensively modified histone [10]. Recent studies have shown that histone modifications can directly regulate the transcriptional processes [11]. Numerous studies have been conducted on H3K9me3 [12, 13]. H3K9me3 is considered to be associated with the repressive states of chromatin [13]. In our study, experiments and analysis were conducted to study the influence of H3K9me3 on the regulation of transcription in ICC. The ICC cell line RBE was sequenced for the histone modification H3K9me3. Based on the results obtained from transcriptomic data analysis (retrieved from TCGA database) and H3K9me3 ChIP-seq data we sequenced, we inferred that H3K9me3 could potentially regulate the abnormal gene expression in ICC and they significantly affected the WNT pathway. The WNT pathway is a signaling pathway that is highly conserved in animals and plants, which regulates cell proliferation, cell migration, differentiation of cells as well as cell cycle [14–16]. The pathway controls numerous processes occurring in tumors. We firstly revealed H3K9me3 may be associated with the WNT pathway as a potential therapeutic target in cholangiocarcinoma.

## Methods

### Cell culture and antibodies

We purchased HIBEpic and RBE cells from Guangzhou Jennio-bio (Guangzhou Jennio-bio Co., Ltd, Guangzhou, China). The cells were cultured at 37 °C in a medium containing 5% CO_2_. All cells were stored in Roswell Park Memorial Institute in a 1640 medium supplemented with 10% fetal bovine serum (FBS; Gibco/BRL, MD, USA), penicillin (100 U/mL), and streptomycin (100 mg/mL) (Beyotime Biotechnology Co., Ltd., Shanghai, China). Sequencing was performed in BGI Shenzhen.

### Western blotting

Total protein from ICC tumor and paracancerous specimens from 10 ICC patients were extracted and separated in 10% SDS-PAGE and then electro-transferred onto polyvinylidene difluoride membranes. After blocking in blocking buffer (Beyotime, China) for 1 h, incubated membranes with primary antibodies (Histone H3 (tri methyl K9), Abcam, ab176916; HistoneH3antibody, Abcam, ab176842;) and secondary antibodies (Goat anti-Rabbit, Proteintech, SA00001-2) were used. Performed the detection of target proteins using enhanced chemiluminescence (ECL) after washing with TBST. All experiments were performed in triplicate.

### ChIP-seq

We performed ChIP experiments using the anti-H3K9me3 antibody (ab8898; Abcam, Cambridge, UK) following previously reported protocols [17]. The cells were crosslinked with formaldehyde and chromatin was fragmented under conditions of sonication. Chromatin from 10^7^ cells was used for each set of ChIP experiments. Protein A Dynabeads (product no. 100.02) were added to chromatin extracts over 2 h under conditions of rotation. Following this, the beads were washed twice with phosphate-buffered saline (1 × PBS). Subsequently, the antibody (2 μg) was added to the beads. We washed the beads and resuspended them in 1 × TE (Tris–EDTA). Following this, the samples were incubated overnight at a temperature of 65 ℃. The NEBNext Ultra DNA Library kit was used to construct the ChIP DNA library. The guidelines provided by the manufacturer (E7370; NEB, Ipswich, MA, USA) were followed to construct the library [18].

### Analysis of ChIP-seq and RNA-sea data from ICC cell line

We used MACS2 (peak-calling tools) to identify the peaks recorded for the RBE and HIBEpic cell lines under conditions of identical parameters [19, 20]. Histone marks such as H3K9me3 are broad. Hence, we set the “broad peak calling” and q-value (adjusted p-value through Benjamini-Hochberg) cut-off at 0.05 [20] (Additional file 1: Table S1). We also considered ChIP-seq peak and Input peak into the same intervals and normalized the reads overlapping the bins by RPKM for both ChIP and Input. Therefore, we could use the foldchange between the bins as a measurement of enrichment over input. In this way, we account for differences in sequencing depth. To filter out the low expression genes, only the genes with TPM > 5 were considered for the analyses. The Homer motif analysis method is used to determine the enriched motif sequences in the peaks [21]. Integrative Genomics Viewer (IGV) was used to visualize the peak abundance tracks [22]. The raw data obtained using the ChIP-seq technique has been deposited in the Sequence Read Archive (SRA) database (https://www.ncbi.nlm.nih.gov/sra) under the accession number SRP333541 (https://www.ncbi.nlm.nih.gov/Traces/study/?acc=SRP333541). To validate our results, other RNA-Seq data for cholangiocarcinoma cell lines were downloaded from the SRA database https://www.ncbi.nlm.nih.gov/Traces/study/?acc=SRP229534&o=acc_s%3Aa).

### TCGA download and analysis

Data on gene expression (level 3), mutation, and clinical data of patients were downloaded from the TCGA (https://tcga-data.nci.nih.gov/) and cBioPortal (https://www.cbioportal.org/) databases [23]. EdgeR (an R package) was used to identify various expressed genes (The significant differently expressed genes was defined as the ones with Benjamini–Hochberg FDR < 0.01 based on all the tests, and |log2(Fold change)|> 2)[24]. The clinical data analysis method was used to analyze the data obtained from cholangiocarcinoma patients. R v3.5.1 was used to perform the analyses. We used the survival data of patients from TCGA database and the overall Kaplan–Meier (KM) survival analysis in each subtype was performed using the “survfit” and “survdiff” functions in the “survival” package [25]. We used GeneMANIA (http://genemania.org) to generate hypotheses on gene function and gene intersection for functional assays [26].

## Results

### Difference in the histone H3K9me3 modifications observed in cholangiocarcinoma and normal cell lines

**Fig. 1:**
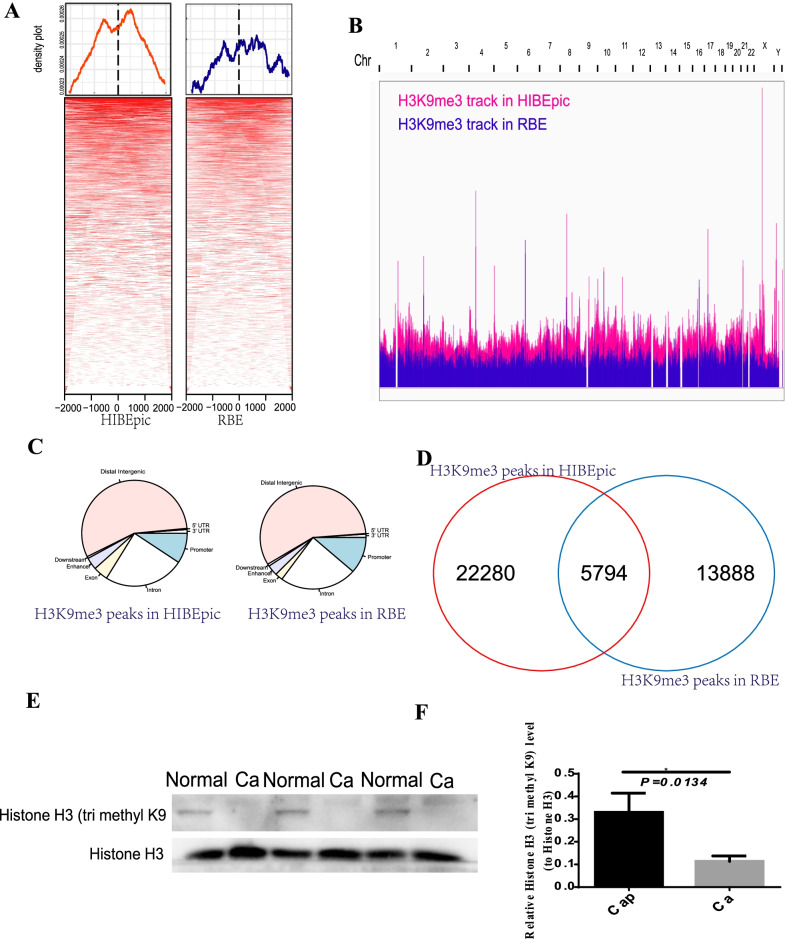
H3K9me3 profiles in RBE and HIBEpic cells. **A** Heatmaps depicting H3K9me3 ChIP-seq peaks enriched at regions spanning TSS sites (± 2 kb) in RBE cells (Right) and HIBEpic cell (Left). **B** Tracks displaying the read coverage of H3K9me3 ChIP-seq normalized by inputs at a whole-genome scale. **C** Distribution of H3K9me3 peaks recorded for RBE and HIBEpic cells. **D** Different H3K9me3 peaks recorded for RBE and HIBEpic represented as Venn diagrams. **E**, **F** H3K9me3 level validated by western blot

We used the ChIP-seq data to analyze histone H3K9me3 present in the RBE and control cell lines. The results revealed that the ChIP peaks of histone H3K9me3 modifications in the ICC cell lines was lesser than that in normal cell lines (Fig. 1A). We also found the number of H3K9me3 peaks of the ICC cell line (RBE) was lesser than that in the normal cell line in a genome-wide scale (Fig. 1B). We annotated the location of identified peaks corresponding to H3K9me3 in the ICC and normal cell lines (Fig. 1C). The number of peaks recorded for RBE was higher by 22,280 when compared to the number of peaks recorded for the normal cell lines (Fig. 1D), suggesting the total number of H3K9me3 peaks decreased in ICC. To further verify our analysis results, we tested H3K9me3 by western blotting to recognize H3K9me3 states, and found the overall H3K9me3 level decreased in ICC patients (Figs. 1E, F, Additional file 2: Fig. S1).

### Analysis of the data obtained from the TCGA database by studying the aberrant expression of cholangiocarcinoma genes

**Fig. 2:**
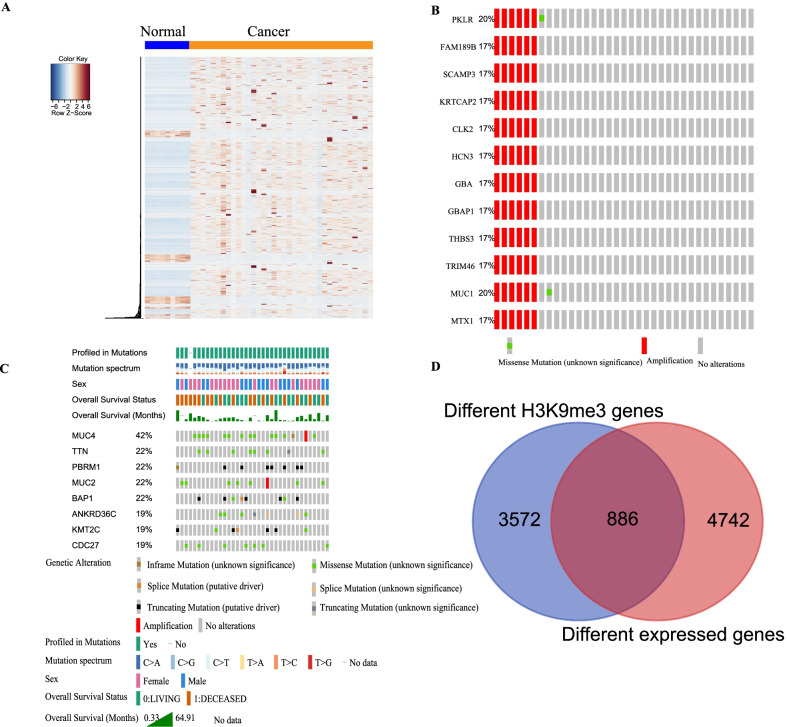
Different expressed genes in ICC. **A** Heatmap and hierarchical clustering tree of differentially expressed genes in CHOL. Rows indicate genes showing significant differences in expression between the normal and cancer tissue of CHOL. **B** Oncoprint showing copy number variation in CHOL. **C** Oncoprint showing the genetic alterations in CHOL. **D** Overlap of H3K9me3 and differentially expressed genes represented as Venn diagrams. **E**–**F** western blotting with antibodies validated to recognize H3K9me3 states

To identify H3K9me3-regulated genes, we identified 5628 differentially expressed genes (Benjamini–Hochberg FDR < 0.01 based on all the tests, and |log2(Fold change)|> 2) by analyzing the RNA-seq data of ICC in TCGA database (Fig. 2A). We observed that most of the differentially expressed genes were highly expressed in ICC tissue. DNA copy number gains occurred more frequently than copy number losses (Fig. 2B, C). These results agreed well with previously reported results that DNA copy number gains occurred more frequently than copy number losses [27]. We analyzed the intersection of the differentially expressed genes and the H3K9me3 histone modifications (specifically absent in the ICC cells) (Fig. 2D). We observed that 886 differentially expressed genes may be potentially regulated by H3K9me3 in ICC (Fig. 2D).

### The relationship of H3K9me3 site and differentially expressed genes in cholangiocarcinoma (CHOL)

**Fig. 3:**
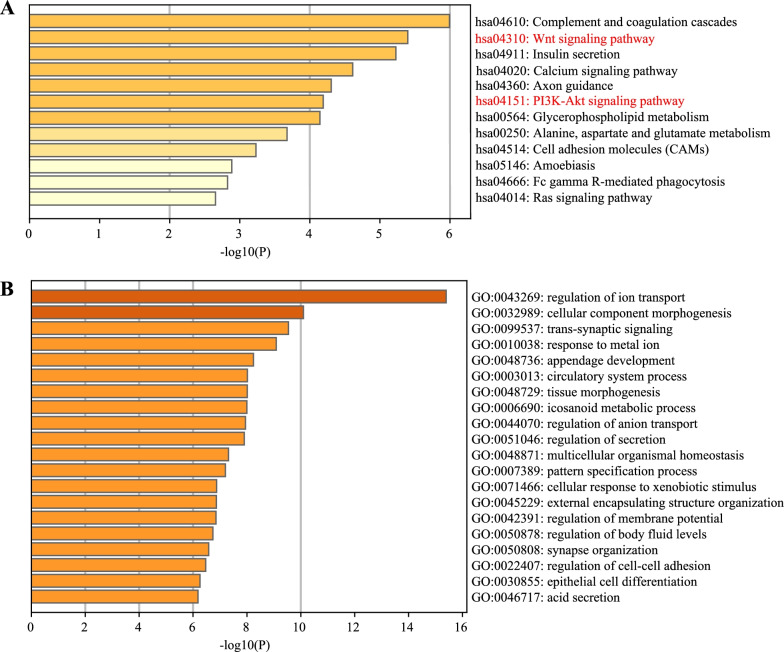
Enrichment analysis of aberrantly and differentially expressed 886 genes. Bar charts representing the KEGG pathway (**A**) and GO (Gene Ontology) (**B**) enrichment analysis of aberrantly and differentially expressed genes

We further explored the biological functions of these 886 differentially expressed genes regulated by H3K9me3. We used gene oncology (GO) analysis and KEGG analysis methods to analyze these differentially expressed genes that were regulated by H3K9me3[28]. We found these overlapped genes were enriched in the WNT pathway, Complement and coagulation cascades, Insulin secretion, Calcium signaling pathway, Axon guidance, PI3K-AKT signaling pathway (Fig. 3A). Therefore, we assumed that WNT pathway is involved in H3K9me3 regulated genes in cholangiocarcinoma. These overlapped genes were enriched in cellular component morphogenesis, regulation of ion transport and trans-synaptic signaling (Fig. 3B). These enriched GO terms are highly related with WNT pathway.

### Clinical features of differentially expressed genes regulated by H3K9me3 in the WNT pathway

**Fig. 4:**
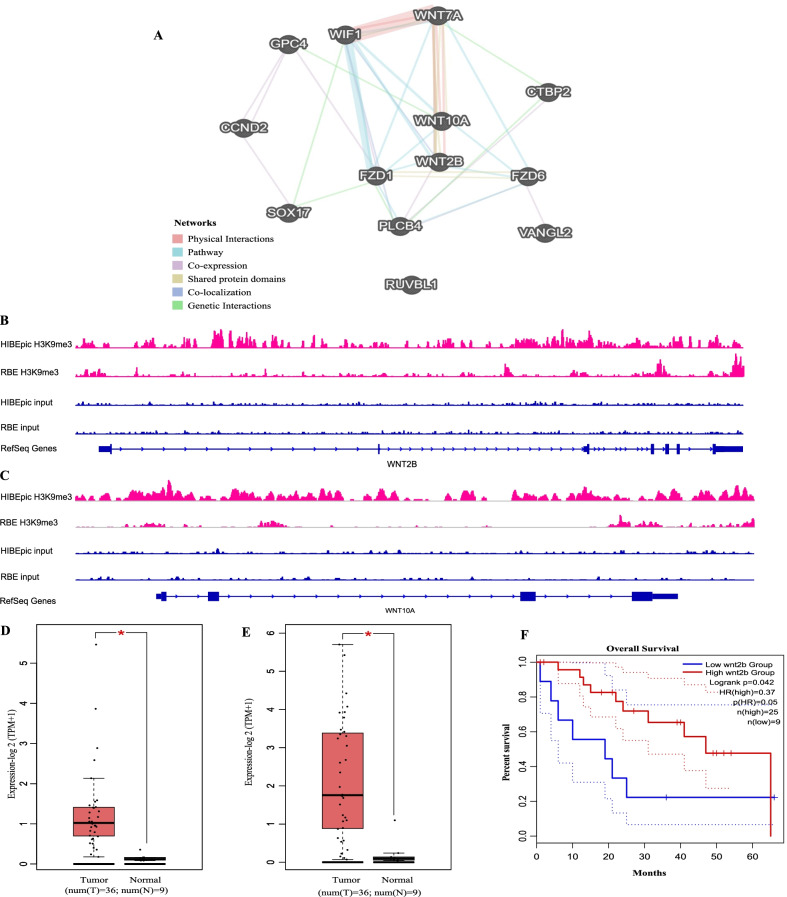
Role of WNT pathway in ICC **A** Gene–gene interaction network among the WNT pathway members. Each node represents a gene, and the inter-node connection lines represent the types of gene–gene interactions. **B**, **C** Integrative Genomics Viewer (IGV) browser shows the tracks of the H3K9me3 peaks in WNT2B and WNT10A. **D**, **E** Boxplot for gene expression level for WNT2B and WNT10A in CHOL. **F** Overall survival curve of WNT2B expression level in ICC patients

To further investigate the association between WNT pathway and H3K9me3-regulated genes. We constructed a network from the differentially expressed H3K9me3-regulated genes that functioned via the WNT pathway by analyzing the data presented in GeneMANIA (Fig. 4A). We identified the core genes (WNT2B and WNT10A) presented in the network. As shown in Fig. 4A, we displayed the WNT-related genes changed in H3K9me3 and gene expression. We observed that the extent of H3K9me3 in WNT2B and WNT10A of the RBE cell line was higher than that in the HIBEpic cell line (Fig. 4B, C). The expression levels of WNT2B and WNT10A in ICC tissue were higher than that in normal tissue (Fig. 4D, E), it is consistent with the result of the extent of H3K9me3 in WNT2B and WNT10A of the RBE cell line. Then, we analyzed the impact of the core gene WNT2B on the prognosis of patients. WNT2B gene also had an effect on the prognosis of ICC patients from TCGA (Fig. 4F).

### Validation of H3K9me3 involved in WNT pathway in ICC using RNAseq data

**Fig. 5:**
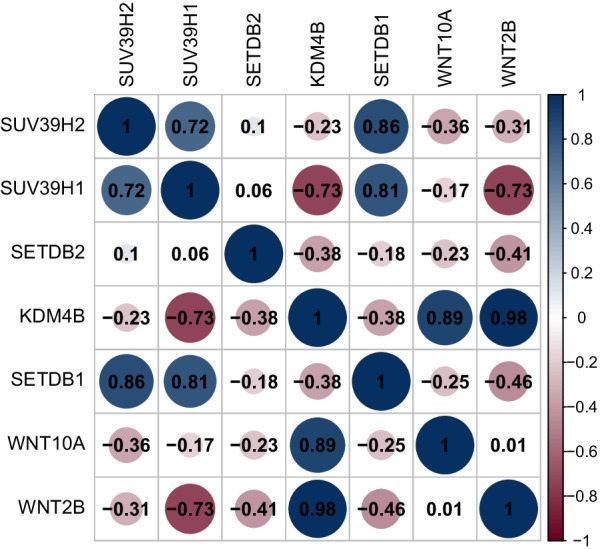
Correlation between expression of H3K9me3 regulators UV39H2, SUV39H1, SETDB2, KDM4B, SETDB1 and expression of WNT2B, WNT10A in CHOL

H3K9me3 was reported to be mainly regulated by SUV39H1, SUV39H2, SETDB1, SETDB2, LSD1 and KDM4B [29, 30]. To further validate our main conclusions that H3K9me3 was involved in the WNT pathway in ICC, we explored the correlations between these H3K9me3’ regulators and core WNT pathway genes—WNT2B and WNT10A using RNA-seq data of ICC cell line and normal cell line. As we expected, the expression levels of WNT2B and WNT10A are associated with the expression level of H3K9me3 regulators (Fig. 5). This is an evidence that H3K9me3 is involved in WNT pathway in ICC.

## Discussion

It has been reported that H3K9me3 significantly influenced the progression of several types of cancers and it could regulate the expression of various cancer-related genes [31–35]. The role of H3K9me3 in regulating genes in cholangiocarcinoma is still unclear. Under conditions of aberrant DNA methylation, H3K9me3 can promote the process of gene silencing [34]. Several histone lysine regulators, such as SUV39H1, SUV39H2, SETDB1, SETDB2, LSD1, EZH2 and KDM4B, significantly influence the process of oncogenesis [29, 36–38]. SUV39H1 and EZH2 has emerged as a drug target and results from studies conducted on SUV39H1 and EZH2 have helped in the development of several potential therapeutic strategies that can be followed to treat cancer [30]. In our study, those regulators were also identified as significantly change gene (Additional file 3: Table S2), indicating H3K9me3 regulators were potential therapeutic target in ICC.

Numerous researchers believed that the initiation of cholangiocarcinoma could be attributed to WNT signaling [39–41]. It is known that WNT signaling can result in cholangiocarcinoma. The initiation occurs when downstream target genes are activated. Researchers have now identified the involvement of the WNT signaling pathway in the differentiation of normal cholangiocytes. Boulter et al. has revealed that the WNT ligands (WNT7B and WNT10A) and β-catenin were highly expressed in the tumor tissues. The interaction between CTBP1 and β-catenin promoted the expression of the WNT target genes [42]. The interactions could be blocked by ICG-001, which was used to study the cholangiocarcinoma model. It was found that ICG-001 significantly hindered the expression of the WNT target genes in cholangiocarcinoma tissues [42]. Under these conditions, a significant reduction in the number of tumors was observed. Therefore, WNT could be considered as a potential therapeutic target in ICC.

## Conclusions

Recently, lots of efforts have been made to develop small-molecule drugs including Porcupine (PORCN) Inhibitor,Tankyrase Inhibitors,TCF/β-Catenin Complex Inhibitors,CBP/β-Catenin Inhibitors,BCL9/β-Catenin Inhibitors,Natural Compounds Target Wnt Signaling Pathway,Challenges to Inhibiting the Wnt/β-Catenin Pathway,to inhibit the WNT pathway for the treatment of many types of cancers[43]. Here, we found that H3K9me3 was involved in regulating gene expression in the WNT pathway in ICC. The simultaneous activity of the EZH2 and WNT inhibitors can potentially help treat ICC. However, further cell biology experimental of the effect of EZH2 and WNT inhibitors might be helpful for arriving at concrete conclusions. Besides, future preclinical experiments that use the mouse and organoids as models would helpful for transformation of our discovery to ICC therapeutic.

## Supplementary Information

**Additional file 1**. Result of H3K9me3 Peaks.

**Additional file 2**. Full length gels and blots with membrane edges.

**Additional file 3**. Differentlly expressed genes in ICC.

## Data Availability

ChIP-seq data generated or analyzed during this study are available in the SRA repository (https://www.ncbi.nlm.nih.gov/Traces/study/?acc=SRP333541).
