# Supplementary material for: Differential enrichment of H3K9me3 in intrahepatic cholangiocarcinoma
Source: BMC Med Genomics. 2022 Aug 26;15:185. doi: 10.1186/s12920-022-01338-1 (PMC9414128; doi:10.1186/s12920-022-01338-1)
Supplement: Supplementary file 2 — Additional file 2. Full length gels and blots with membrane edges. [file 12920_2022_1338_MOESM2_ESM.pptx]

## Slide 1
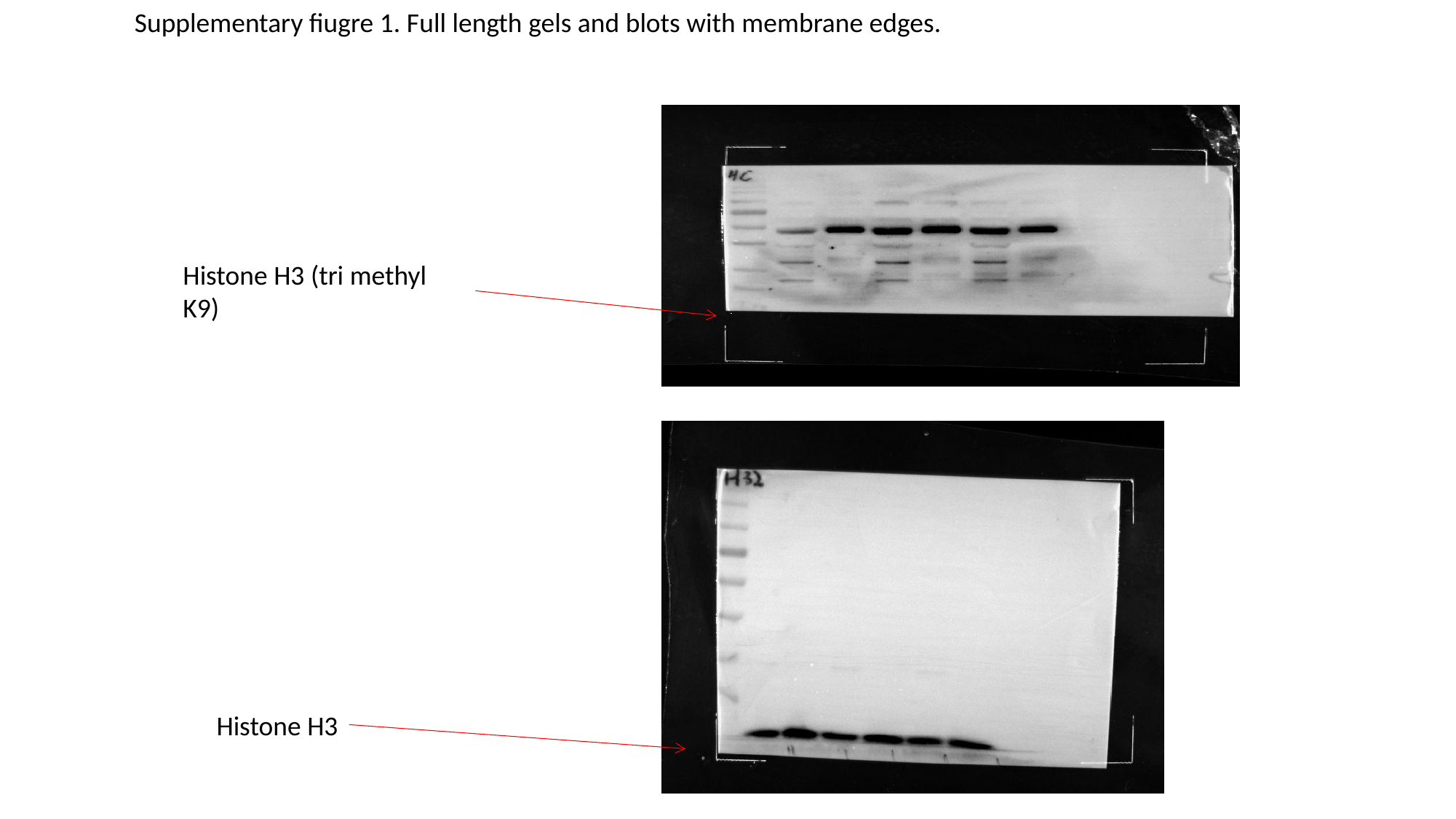

Supplementary fiugre 1. Full length gels and blots with membrane edges.
Histone H3 (tri methyl K9)
Histone H3
